# Pubertal timing in boys and girls born to mothers with gestational diabetes mellitus: a systematic review

**DOI:** 10.1530/EJE-20-0296

**Published:** 2020-10-08

**Authors:** Anuradhaa Subramanian, Jan Idkowiak, Konstantinos A Toulis, Shakila Thangaratinam, Wiebke Arlt, Krishnarajah Nirantharakumar

**Affiliations:** 1 Institute of Applied Health Research, University of Birmingham, Birmingham, UK; 2 Institute of Metabolism and Systems Research, University of Birmingham, Birmingham, UK; 3 Centre for Endocrinology, Diabetes and Metabolism, Birmingham Health Partners, University Hospitals Birmingham NHS Foundation Trust, Birmingham, UK; 4 Department of Endocrinology and Diabetes, Birmingham Children’s Hospital, Birmingham Women’s and Children’s NHS Foundation Trust, Birmingham, UK

## Abstract

**Context:**

The incidence of gestational diabetes mellitus (GDM) has been on the rise, driven by maternal obesity. In parallel, pubertal tempo has increased in the general population, driven by childhood obesity.

**Objective:**

To evaluate the available evidence on pubertal timing of boys and girls born to mothers with GDM.

**Data sources:**

We searched MEDLINE, EMBASE, CINAHL Plus, Cochrane library and grey literature for observational studies up to October 2019.

**Study selection and extraction:**

Two reviewers independently selected studies, collected data and appraised the studies for risk of bias. Results were tabulated and narratively described as reported in the primary studies.

**Results:**

Seven articles (six for girls and four for boys) were included. Study quality score was mostly moderate (ranging from 4 to 10 out of 11). In girls born to mothers with GDM, estimates suggest earlier timing of pubarche, thelarche and menarche although for each of these outcomes only one study each showed a statistically significant association. In boys, there was some association between maternal GDM and earlier pubarche, but inconsistency in the direction of shift of age at onset of genital and testicular development and first ejaculation. Only a single study analysed growth patterns in children of mothers with GDM, describing a 3-month advancement in the age of attainment of peak height velocity and a slight increase in pubertal tempo.

**Conclusions:**

Pubertal timing may be influenced by the presence of maternal GDM, though current evidence is sparse and of limited quality. Prospective cohort studies should be conducted, ideally coupled with objective biochemical tests.

## Introduction

Puberty marks an important period in the dynamics of childhood development characterised by fundamental physical, cognitive and psychological transformation. The attainment of adult-like secondary sexual characteristics, rapid growth, changes of body composition and achieving fertility are the main physical outcomes of puberty. As a consequence of the maturation of the hypothalamic–pituitary–gonadal axis with subsequent incremental, finely orchestrated gonadal sex steroid production, typical physical changes occur in a successive fashion. In girls, this usually starts with thelarche (onset of breast development) and pubarche (appearance of pubic hair), followed by a peak growth spurt culminating in menarche (first menstruation) (1). In boys, testicular enlargement and pubarche are the first physical signs of puberty followed by peak growth spurt and spermarche (development of sperm) with the occurrence of the first ejaculation.

A secular trend of advancement in pubertal timing along with a steep decline in the age of menarche from 17 to 13 years has been recognized between 19th and 21st century (1, 2, 3). Consequently, increasing numbers of children are diagnosed with central precocious puberty (1, 2), defined as the onset of gonadarche before the age of 8 years in girls and 9 years in boys, a definition based upon assessment of pubertal staging performed by Tanner *et al*. in a large cohort of children in the 1960s (4, 5). Compared to peers who mature on time or later, early developers are more likely to experience psychological distress and social isolation, potentially leading to detrimental outcomes such as poor academic performance, depression, substance abuse, eating disorder, disturbed body image and risky sexual behaviour (6, 7). Early pubertal timing also has an adverse impact on adult metabolic health including increased risk of diabetes and other cardio-vascular morbidity (6, 7, 8).

Risk factors for early puberty are considered to be multifactorial and may be seen as the effect of factors influencing the maturation of the hypothalamic GnRH pulse generator. These include predisposing genetic factors, intrauterine environment, and endocrine-disrupting chemicals, and, first and foremost, abundance of nutrients and childhood obesity (9). Similar to the trend towards earlier pubertal timing driven by childhood obesity, the incidence of gestational diabetes mellitus (GDM) driven by maternal obesity has also been on the rise; in some countries, the incidence of GDM has doubled in the last decade and is predicted to further increase (10), although changes in screening practices might have contributed to this rise (11).

The effect of maternal GDM on pre-pubertal health outcomes in the offspring has been evaluated by a limited number of observational studies, but evidence on the effect of GDM on sexual maturation and pubertal timing is scarce and conflicting. Due to the complexity in the conceptualization of pubertal timing and its clinical assessment and the significant heterogeneity among the studies exploring the relationship between maternal GDM and central precocious puberty, a causal relationship has not been clearly established yet. If confirmed, such a link could drive a transgenerational continuum and, thereby, metabolic morbidity associated with both conditions. Here, we have undertaken a systematic appraisal of the available evidence on pubertal timing in children born to mothers with GDM.

## Methods

### Searches

We carried out a systematic literature review search initially in March 2019, with the search rerun in October 2019 to retrieve any additional studies before final synthesis of results. Databases included: (i) Electronic bibliographic databases (MEDLINE, EMBASE, CINAHL Plus, Cochrane library), (ii) Google Scholar™ search and experts contact to obtain relevant grey literature, and (iii) citations tracked from the screened articles to identify further relevant studies. The search strategy was constructed with the help of a medical librarian combining natural and structured language terms (MESH and Emtree). Terms relating to ‘gestational diabetes’ was combined with an ‘AND’ Boolean operator to ‘puberty’, ‘pubarche, ‘thelarche’, ‘menarche’, ‘Tanner staging’, ‘spermarche’ and ‘growth’. A list of search terms is provided in Supplementary Table 1 (see section on supplementary materials given at the end of this article).

Records identified by the searches were independently screened by two reviewers (A.S. and J.I.) in the order of title, abstract and full text of the article. Articles were selected when they met the inclusion and exclusion criteria mentioned in the pre-defined protocol registered on PROSPERO (CRD42019150365). In case of study selection disagreements, a third reviewer (K.N.) was consulted to reach consensus.

### Inclusion and exclusion criteria

We included observational studies – cohort, case–control and cross-sectional studies. Studies that considered multiple exposures or multiple outcomes were also included, if they studied the association between maternal GDM and pubertal timing in the offspring. Pubertal timing was allowed to be described by the timing of the following pubertal milestones according to Tanner (4, 5): in girls, (i) pubic hair development/pubarche (Tanner stage: ≥PH2), (ii) breast development/thelarche (Tanner stage: ≥B2), (iii) menarche and (iv) speed of pubertal growth as peak height velocity (PHV) and age at PHV; in boys, (i) pubic hair development/pubarche (Tanner stage: ≥PH2), (ii) testicular enlargement (testicular volume ≥4 mL on either or both sides), (iii) maturation of the external male genitalia (Tanner stage: ≥G2), (iv) spermarche and (v) PHV and age at PHV.

Studies were excluded if they were case studies, case series or commentary articles, qualitative studies without quantitative data on pubertal timing, studies reporting pubertal staging instead of pubertal timing disregarding chronological age, or studies conducted on non-human subjects.

### Data extraction and risk of bias assessment

The JBI data extraction form (12) was adapted based on the specifics of this review to create a template form in Microsoft Word® (Supplementary Table 2). The form mandated data on the following elements from the included studies: authors, study publication date, data source, study period, country and setting, sample size, GDM exposure ascertainment criteria, proportion of GDM exposed women who used insulin, offspring sex, outcome/s considered and details on analytical methods employed including the list of confounding variables considered.

An adapted version of the Newcastle–Ottawa critical appraisal checklist (13) was used to evaluate the risk of bias of each of the included studies and individual studies were graded as low or high risk for each of the checklist questions (template form is provided in Supplementary Table 3). Elements employed in appraising the internal validity of the included studies included: (i) potential selection bias, that is, inclusion criteria or study setting giving rise to systematic difference of the sampled cohort from the general population; (ii) objective GDM diagnosis and pubertal staging measurement; (iii) capture of and adjustment for confounding variables; (iv) appropriateness of statistical analysis employed to account for uncertainty in the true event time such as interval censored time-to-event analysis or modelling multiple longitudinal outcome records; and (v) sufficient follow-up period and characteristics of patients lost to follow-up. Representativeness of the study population was also discussed to assess external validity.

Data extraction and risk of bias assessment forms were pilot-tested with one of the included studies at the protocol-writing stage. Data extraction and quality appraisal were performed by two independent reviewers (A.S. and J.I.) and in case of disparities, a third reviewer (K.N.) was contacted to settle differences.

Findings of this review are reported in accordance with PRISMA guidelines (Supplementary Table 4) (11).

## Results

### Literature search results

**Figure 1:**
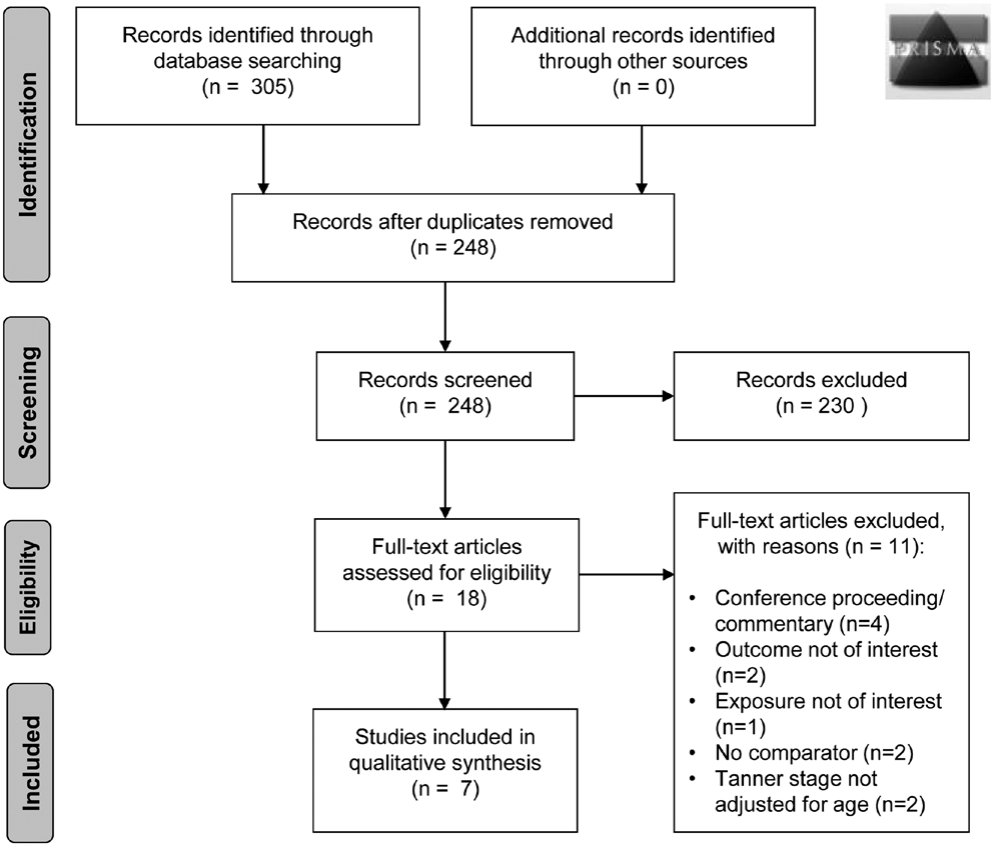
PRISMA flowchart.

We identified 305 studies through electronic database searches, including 57 duplicates (Fig. 1). Of the remaining 248, 230 were not relevant to the research question and were excluded on the basis of title and abstract, leaving 18 studies for full-text assessment. Eleven articles were excluded at this stage: four articles were conference proceedings, oral presentations or commentary articles (14, 15, 16, 17); two articles did not include any of the outcomes we were interested in (18, 19); one article did not analyse GDM as a predictor for pubertal timing due to an insufficient number of subjects with GDM (20); two articles did not provide a comparator cohort (21, 22); two articles only reported Tanner stage at baseline and did not consider age/timing of puberty (23, 24). The seven remaining studies were included in the review (Fig. 1).

### Study characteristics

The seven primary studies included in this review (25, 26, 27, 28, 29, 30, 31) are described in Table 1. Four studies were conducted in the USA (25, 27, 28, 29), two in Denmark (26, 30) and one in England (31). The populations studied were predominantly Caucasian. Four studies had comparable primary objectives to our review question (27, 28, 29, 30) two studies looked at multiple predictors of pubertal timing (25, 31), and one looked at multiple developmental outcomes in the offspring of mothers with GDM including pubertal timing (26).

**Table 1:** Characteristics of the included studies.

No.	Reference	Location	Study database	GDM diagnostic criteria	Age of mothers^¶,@^	Offspring sex	Sample size		Outcomes considered
							GDM	Controls	
1	(26)^†^	USA and Puerto Rico	Sister study	Self reports from family history questionnaires completed by the offspring	^‡^<20 years: 1537 (5%); 20–34 years: 25191 (76%); ≥35 years: 6380 (19%)	Girls	178	30 169	Adjusted RR for age at menarche: ≤ 10, 11, 12–13 (reference age group), 14 and ≥ 15 years
2	(27)	Denmark	DNBC	DNPR criteria based on WHO criteria* + self reports	NR	Girls	238	256	Age adjusted OR for ≥ Tanner B2
							221	237	Age adjusted OR for ≥ Tanner PH2
					NR	Boys	192	182	Age adjusted OR for testicular volume ≥ 4 mL
							206	203	Age adjusted OR for ≥ Tanner PH2
							193	176	Age adjusted OR for ≥ Tanner G2
3	(28)	USA	KPCO linked to EPOCH study	NDDG criteria**	NR	Girls	34§	174	Age at PHV stratified by exposure status; PHV stratified by exposure status. β coefficients and *P*-value from accelerated failure time model and *P*-value.
					NR	Boys	43§	166	Age at PHV stratified by exposure status; PHV stratified by exposure status. and beta coefficients and *P*-value from accelerated failure time model and *P*-value
4	(29)	USA	KPNC linked to CYGNET	Carpenter and Coustan criteria***	^¶^GDM: 33.0 (5.0); Control: 32.6 (5.9)	Girls	27	390	Adjusted HR: ≥ Tanner B2 ≥ Tanner PH2
5	(30)^†^	USA	KPNC	Carpenter and Coustan criteria***	^¶^UW:27.9(5.2) ; NW: 29.9(5.1); OW: 30.5(5.2); OB: 30.3(5.3)	Girls	977	11 364	Adjusted HR for ≥ Tanner B2
							931	10 839	Adjusted HR for ≥ Tanner PH2
6	(31)	Denmark	DNBC linked to the Puberty Cohort	DNPR based on WHO criteria* + self reports	^@^GDM: 31.9 (31.4–32.5) Control: 30.6 (30.5–30.7)	Girls	122	15 320	Adjusted mean monthly difference for: Tanner B2, B3, B4, B5 Tanner PH2, PH3, PH4, PH5 Menarche Axillary hair Acne
						Boys	130		Adjusted mean monthly difference for: Tanner G2, G3, G4, G5 Tanner PH2, PH3, PH4, PH5 Spermarche Voice break Adult Voice Axillary hair Acne
7	(32)^†^	UK	Avon Longitudinal Study of Parents and Children	Self reports	^¶^29.0 (3.8)	Boys	450	3263	Median age at transition to: Tanner PH > 1, PH > 2 and PH > 3 based on multi-stage modelling

^†^Summarises the cohort from the primary study (potential contamination from mothers with pre-existing diabetes or patients with missing information on GDM); Data presented as ^‡^*n* (%); ^¶^Mean (s.d.); ^@^Median (IQR); ^§^10% contamination of the GDM exposed cohort with exposure to type 1 diabetes; *Danish National Patient Registry Criteria: GDM diagnosis is made based on WHO criteria (see subsequently) and in some cases if two or more glucose values measured exceeded the mean ± 3 s.d. on a curve based on a group of 40 healthy, nonobese, nonpregnant Danish women without a family history of diabetes, WHO criteria: fasting blood glucose > 7.0 mmol/L or random blood glucose > 11.1 mmol/L or 75 g oral glucose tolerance test at 2 h > 11.1 mmol/L; **NDDG criteria: 50 g oral glucose tolerance test at 1 h ≥7.8 mmol/L followed by a 100 g oral glucose tolerance test at 3 h > 7.8 mmol/L; ***Carpenter and Coustan criteria: fasting blood glucose > 5.3 mmol/L or 100 g oral glucose tolerance test at 1 h > 10 mmol/L, at 2 h > 8.3 mmol/L, at 3 h > 7.8 mmol/L.

CYGNET, Cohort Study of Young Girls’ Nutrition, Environment and Transitions; DNBC, Danish national Birth cohort; DNPR, Danish National Patient Registry; EPOCH, Exploring Perinatal Outcomes among Children; HR, Hazard Ratio; KPCO, Kaiser Permanente of Colorado Health Plan; KPNC, Kaiser Permanante Northern California; NDDG, National Diabetes Data Group; NR, not reported; NW, normal weight; OB, obese; OR, Odds Ratio; OW, overweight; PHV, peak height velocity; RR, Risk Ratio; UW, underweight.

Three of the included studies focussed only on the pubertal timing in girls (25, 28, 29), one study focussed only on the pubertal timing in boys (31) and three studies reported outcomes for both boys and girls (26, 27, 30). All of the studies stratified their estimates by offspring sex.

Two pairs of the included articles derived their study sample from the same pregnancy cohorts and thus had the potential for overlapping populations (Danish National Birth Cohort (DNBC) (26, 30) and Kaiser Permanante Northern California (KPNC) (28, 29)).

Sample size ranged widely both between and within studies when considering multiple outcomes (Table 1): D’Aloissio *et al.* included 33 501 daughters with information on age at menarche; 178 of them self-reported positive maternal GDM status through telephone contact with their mothers (25). Grunnet *et al.* considered multiple outcomes: breast and pubic hair development in 494 and 458 girls, respectively, and testicular volume, pubic hair, and genital developmental stage in 374, 409 and 369 boys, respectively (26). Hockett *et al.* included 208 girls and 209 boys with anthropometric records to calculate peak height velocity; 34 girls and 43 boys had positive maternal GDM status (27). Two studies that used the same cohort (KPNC) and considered the same outcomes varied with regard to the maternal sample size (417 and 12 341) (28, 29). Lauridsen *et al.* included 122 and 130 girls and boys with positive maternal GDM exposure status (30), while Monteilh *et al.* included 450 boys with positive maternal GDM exposure status (31).

### Risk of bias assessment

**Figure 2:**
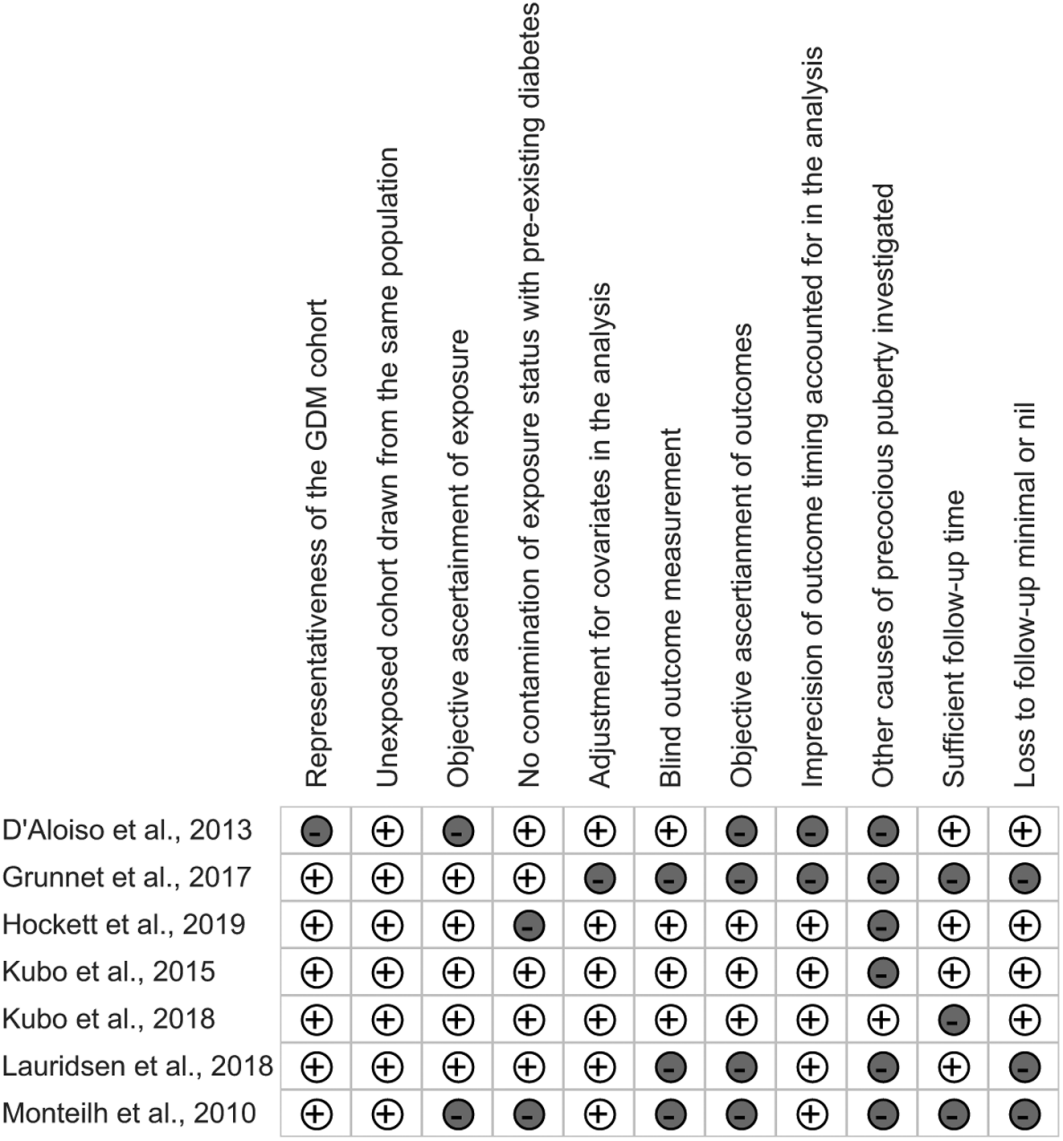
Assessment of risk of bias.

The risk of bias based on the review question-adapted Newcastle Ottawa critical appraisal checklist is summarized for the seven included studies in Fig. 2. All populations studied were reasonably representative of their respective country’s general practice or hospital setting except for the study by D’Aloisio *et al.* (25), who had restricted inclusion to pregnant women at risk of breast cancer. Exposure information regarding GDM status was obtained from pregnancy registries in five studies (26, 27, 28, 29, 30), two of those studies also considered self-reports (26, 30). However, for the remaining two studies (25, 31), GDM status was only self-reported, indicating high risk of recall or misclassification bias. Studies based on KPNC cohorts mentioned using Carpenter and Coustan thresholds for GDM diagnosis. Variation was observed in the covariates considered, with race/ethnicity and socio-economic status representing the most popular confounders considered in the association between maternal GDM and pubertal timing in the offspring.

Outcome measurements were performed by research staff in four studies (26, 27, 28, 29); three of them specifically reported utilization of recommended methods to measure outcomes, such as orchidometer use for the assessment of testicular size, breast palpation for accurate assessment of the stage of breast development, and computational modelling (Superimposition by Translation And Rotation (SITAR)) of longitudinal height measurements for PHV and age at PHV (27, 28, 29). Outcomes were recorded only during a series of pre-defined observation times prohibiting the capture of precise pubertal timing, but four studies performed interval censoring to account for this in their analysis (28, 29, 30, 31). Notably, two studies recorded Tanner stage in less than 80% of the offspring (30, 31), suggesting a possibility of dropout bias.

### Association between maternal gestational diabetes and pubertal timing in girls

Results of the primary studies reviewing the association between maternal GDM and pubertal onset in girls, as indicated by age at menarche, pubarche and thelarche, are given in Table 2.

**Table 2:** Evidence summary of the relationship between maternal GDM and pubertal development in their daughters identified by pubic hair development (*n* = 4 studies), breast development (*n* = 4), peak height velocity (*n* = 1), menarche (*n* = 2) and other pubertal changes (*n* = 1).

Outcome/study	Outcome metrics	Estimates
Pubic hair development/pubarche		
(27)	*n* (%) of ≥ Tanner PH2 in GDM and controls Age adjusted OR (95% CI) of ≥ Tanner PH2	GDM: 99 (44.8%); Controls: 133 (56.1%); OR, adjusted: 1.51 (0.90, 2.55); *P* = 0.12
(30)	Adjusted (for race/ethnicity, maternal age, education, parity, smoking during pregnancy and BMI) HR (95% CI) of ≥ Tanner PH2	HR adjusted: 1.04 (0.92, 1.17)
(31)	Mean age at Tanner PH2, PH3, PH4, PH5 in daughters of mothers without diabetes	Mean Age (years): PH2: 11.3; PH3: 12.5; PH4: 13.4; PH5: 15.3
	Crude and adjusted (for maternal age at menarche, maternal age at birth, socioeconomic status, cohabitation, parity and maternal BMI) mean monthly difference in age (95% CI) at Tanner PH2, PH3, PH4, PH5	Mean difference (months): PH2: crude: −5.4; adjusted: −4.8 (−7.7, −2.0); PH3: crude: −3.1; adjusted: −2.2 (−4.4, 0); PH4: crude: −2.6; adjusted: −1.6 (−4.8, 1.6); PH5:crude: −7.8; adjusted: −6.0 (−10.8, −1.2);
(29)	Unadjusted and adjusted (for race/ethnicity, household income and maternal age) hazard ratio (95% CI) of ≥ Tanner PH2	HR: crude: 1.09 (0.71, 1.70); adjusted: 1.24 (0.79, 1.94)
	Similar estimates for the interaction between maternal pregravid BMI and GDM:	HR, adjusted: BMI < 25 and no GDM: Reference category; BMI < 25and GDM: 1.06 (0.48, 2.36); BMI ≥ 25 and no GDM: 1.19 (0.90, 1.56); BMI ≥ 25 and GDM: 2.97 (1.52, 5.83)
Breast Development/Thelarche		
(27)	*n* (%) ≥ Tanner B2 in GDM cases and controls	GDM: 141 (59.2%); controls: 169 (66.0%)
	Age adjusted OR (95% CI) of ≥ Tanner B2	OR adjusted: 1.99 (1.18, 3.34); *P* = 0.01
		Estimate with additional adjustment for BMI shown in primary study’s figure (direction of effect remains but statistically NS)
(30)	Adjusted (for race/ethnicity, maternal age, education, parity, smoking during pregnancy and BMI) hazard ratio (95% CI) of ≥ Tanner B2	HR adjusted: 1.06 (0.95, 1.18)
(31)	Mean age at Tanner stage B2, B3, B4, B5 in daughters of mothers without diabetes	Mean age (years): B2: 9.9; B3: 11.6; B4: 13.0; B5: 15.7
	Crude and adjusted (for maternal age at menarche, maternal age at birth, socioeconomic status, cohabitation, parity and maternal BMI) mean monthly difference in age (95% CI) at Tanner stage B2, B3, B4, B5	Mean difference (months): B2: crude: −7.3; adjusted: −4.6 (-10.1, 1.0); B3: crude: −3.8; adjusted: −1.9 (−5.0, 1.2) B4: crude: −2.1; adjusted: −0.5 (−3.2, 2.4) B5: crude: −4.3; adjusted: −1.8 (−7.9, 4.3)
(29)	Unadjusted and adjusted (for race/ethnicity, household income and maternal age) hazard ratio (95% CI) of breast development stage ≥ B2	HR: crude: 1.01 (0.65, 1.57); adjusted: 0.85 (0.54, 1.35)
	Similar estimates for pregravid BMI # GDM interaction	HR adjusted: BMI < 25#No GDM: Reference category; BMI < 25#GDM: 1.22 (0.54, 2.74); BMI ≥ 25#No GDM: 1.18 (0.88, 1.57); BMI ≥ 25#GDM: 0.93 (0.47, 1.85)
PHV and age at PHV		
(28)	Age at PHV stratified by maternal GDM exposure status and beta coefficient for exposure to GDM in utero after adjusting for child’s race/ethnicity	Age at PHV (years): GDM: 10.85; No GDM: 11.12 β coefficient and *P*-value not reported (but figure shows overlapping confidence intervals of age at PHV between exposed and the unexposed)
	PHV among exposed and unexposed girls and boys and beta coefficient for exposure to GDM in utero after adjusting for child’s race/ethnicity	PHV (cm/year): GDM: 8.88; No GDM: 8.04; β coefficient: 0.10; *P <* 0.001
Menarche		
(26)	RR(95% CI) of earlier or later menarche in mothers with GDM compared to mothers without GDM after adjustment for birth decade, race/ethnicity, childhood family income, interaction between birth decade and race/ethnicity	RR adjusted: ≤ 10 years: 0.98 (0.53, 1.84); 11 years: 0.99 (0.63, 1.54); 12–13 years: reference category; 14 years: 0.77 (0.48, 1.25); ≥ 15 years: 0.98 (0.60, 1.60)
(31)	Mean age at menarche in daughters of mothers without diabetes	Mean age (years): 13.0
	Crude and adjusted (for maternal age at menarche, maternal age at birth, socioeconomic status, cohabitation, parity and maternal BMI) mean monthly difference in age (95% CI) at menarche	Mean difference (months): crude: −4.1; adjusted: −2.5 (−4.9, 0.0)
Other pubertal outcomes		
(31)	Crude and adjusted (for maternal age at menarche, maternal age at birth, socioeconomic status, cohabitation, parity and maternal BMI) mean monthly difference in age (95% CI) at development of axillary hair and acne	Mean difference (months): AH: crude: −4.4; adjusted: −3.6 (−7.3, 0.1) Acne: crude: −3.8; adjusted: −2.6 (−6.8, 1.6)

AH, axillary hair; B, breast development; HR, hazard ratio; NS, not significant; OR, odds ratio; PH, pubic hair; PHV, peak height velocity; RR, risk ratio.

#### Pubic hair development/pubarche

There was an inconsistent association between maternal GDM and pubarche in girls based on the four primary articles that studied this association. Lauridsen *et al.* (30) reported an earlier age at attainment of all pubic hair stages in girls of mothers with GDM ranging between 1.6 and 6.0 months after adjustment (adjusted mean monthly difference in PH2: −4.8 (95% CI: −7.7, −2.0); PH3: −2.2 (95% CI: −4.4, 0); PH4: −1.6 (95% CI: −4.8, 1.6); PH5: −6.0 (95% CI: −10.8, −1.2)) (Table 2). Three studies considered pubertal Tanner stages of ≥PH2 as an outcome (26, 28, 29). Grunnet *et al*. (26) reported an increase of 51% in age adjusted odds for reaching ≥PH2 in girls born to mothers with GDM (adjusted OR: 1.51 (95% CI: 0.90, 2.55)) (Table 2). Kubo *et al.* conducted two studies in 2016 (28) and 2018 (29) using datasets derived from the same database assessing the hazard ratio to reach ≥PH2 for girls of mothers with GDM compared to controls (2015 study adjusted HR: 1.04 (95% CI: 0.92, 1.17) and 2018 study adjusted HR: 1.24 (95% CI: 0.79, 1.94)) (Table 2). When accounting for interaction between maternal pregravid BMI and GDM, there was a 3-fold increased hazard of Tanner stage ≥PH2 among girls born to mothers with GDM and a pregravid BMI ≥25 compared to mothers without GDM and a pregravid BMI <25 (adjusted HR: 2.97 (95% CI: 1.52, 5.83)).

#### Breast development/thelarche

The same four studies that studied the association between pubarche and maternal GDM also studied the association between breast development and GDM (26, 28, 29, 30). Lauridsen *et al.* (30) reported the mean age at Tanner breast stages 2–5 in girls born to mothers with and without GDM. The direction of effect size suggest a lower age for all Tanner stages among girls born to mothers with GDM (adjusted mean monthly difference in B2: −4.6 (95% CI: −10.1, 1.0); B3: −1.9 (95% CI: −5.0, 1.2); B4: −0.5 (95% CI: −3.2, 2.4); B5: −1.8 (95% CI: −7.9, 4.3)) (Table 2). Grunnet *et al.* (26) showed twice the age adjusted odds of ≥B2 among girls born to GDM mothers compared to controls (age adjusted OR: 1.99 (95% CI: 1.18, 3.34)) (Table 2); however, once adjusted for offspring BMI, the significance in this association was no longer evident. The association between maternal GDM and offspring age at thelarche in the 2015 and 2018 studies conducted by Kubo et al. was not evident (adjusted HR: 0.85 (0.54–1.35) and 1.06 (0.95–1.18), respectively) (28, 29) (Table 2).

#### Age at peak height velocity

Hockett *et al.* (27) examined the association between maternal GDM and pubertal timing in the daughters as reflected by growth parameters including peak height velocity (PHV) and age at PHV (APHV). APHV was 10.85 years in girls born to mothers with GDM and 11.12 years in girls born to mothers without GDM, with overlapping confidence intervals (Table 2). Using a log-logistic accelerated failure time model, daughters born to mothers with GDM had a 10% higher ethnicity-adjusted height velocity than girls born to mothers without GDM (Table 2).

#### Menarche

Maternal GDM seemed to be associated with earlier age at menarche but the evidence is inconsistent. D’Aloisio *et al.* (25) found that girls born to mothers without pre-gestational or gestational diabetes had no increased risk of earlier (≤10 and 11 years) or later age at menarche (14 and ≥15 years) in comparison to an arbitrary defined reference age of 12–13 years after adjusting for birth decade, ethnicity and family income (Table 2). By contrast, girls born to mothers with pregnancy hyperglycemia had a significantly higher risk of earlier menarche (≤10 years) (adjusted RR: 1.47 (95% CI: 1.01, 2.16)). In keeping with these findings, Lauridsen *et al.* (30) report a significant earlier onset of menarche by 2.5 months in girls born to mothers with GDM compared to mothers without diabetes (adjusted mean monthly difference: −2.5 (95% CI: −4.9, 0)) (Table 2).

### Association between maternal gestational diabetes and pubertal timing in boys

Results of the primary studies reviewing the association between maternal GDM and pubertal onset exclusively among boys indicated by age at spermarche, pubarche and genital development are shown in Table 3.

**Table 3:** Evidence summary: The relationship between maternal GDM and pubertal development in their sons identified by pubic hair development (*n* = 3 studies), testicular development (*n* = 2), genital development (*n* = 2), peak height velocity (*n* = 1), spermearche (*n* = 1) and other pubertal changes (*n* = 1).

Outcome/study	Outcome metrics	Estimates
Pubic hair development/pubarche		
(27)	*n* (%) of ≥ Tanner stage PH2 in GDM cases and controls	GDM: 50 (24.3%); Controls: 60 (29.6%)
	Age adjusted OR (95% CI) of ≥ Tanner stage PH2	OR adjusted: 1.74 (0.92, 3.28); *P* = 0.09
		Estimate with additional adjustment for BMI shown in primary study’s figure (direction of effect remains and still statistically NS)
(31)	Mean age at Tanner stage PH2, PH3, PH4, PH5 in sons of mothers without diabetes	Mean age (years): PH2: 11.3; PH3: 12.7; PH4: 13.5; PH5: 14.7
	Crude and adjusted (for maternal age at menarche, maternal age at birth, socioeconomic status, cohabitation, parity and maternal BMI) mean monthly difference in age (95% CI) at Tanner stage PH2, PH3, PH4, PH5	Mean difference (months): PH2 crude: −1.9; adjusted: −1.4 (−5.3, 2.4); PH3 crude: −1.9; adjusted: −1.3 (−4.6, 1.9); PH4 crude: −1.7; adjusted: −0.8 (−3.4, 1.6); PH crude: −2.6; adjusted: −1.7 (−4.7, 1.3)
(32)	Multistage modelling: Median age at transition (95% CI) to >Tanner stage PH1, >Tanner stage PH2, >Tanner stage PH3	PH>1: GDM was not a covariate in the combined multivariate model level due to statistical insignificance at the univariate analysis or restricted combined model level
		PH > 2: In the model with offspring BMI at age 8, Median age at PH > 2 (years): 12.8 (12.7–12.8) Median age at PH > 2 among sons of GDM mothers (years): 12.6 (12.4–12.7); *P* = 0.03
		In the model with offspring height and weight at age 8, Median age at PH > 2 (years): 13.0 (12.8–13.1) Median age at PH > 2 in sons of GDM mothers (years): 12.8 (12.6–13.0); *P* = 0.05
		PH > 3: GDM was not a covariate in the combined multivariate model level due to statistical insignificance at the univariate analysis or restricted combined model level
Testicular development		
(27)	*n* (%) of testicular volume ≥4 mL in GDM cases and controls	GDM: 143 (74.5%); No GDM: 156 (85.7%)
	Age adjusted OR (95% CI) of testicular volume ≥4 mL	OR, adjusted: 0.77 (0.42–1.41); *P* = 0.40
		Estimate with additional adjustment for BMI shown in primary study’s figure (direction of effect remains and still statistically NS)
Genital Development		
(27)	*n* (%) of ≥ Tanner stage G2 in GDM cases and controls	GDM: 63 (32.6%); No GDM: 66 (37.5%)
	Age adjusted OR (95% CI) of ≥ Tanner stage G2	OR Adjusted: 1.24 (0.72, 2.14); *P* = 0.45
		Estimate with additional adjustment for BMI shown in primary study’s figure (Direction of effect remains and still statistically NS)
(31)	Mean age at Tanner stage G2, G3, G4, G5 in sons of mothers without diabetes	Mean age (years): G2: 10.9; G3: 12.5; G4: 13.6; G5: 15.5
	Crude and adjusted (for maternal age at menarche, maternal age at birth, socioeconomic status, cohabitation, parity and maternal BMI) mean monthly difference in age (95% CI) at Tanner stage G2, G3, G4, G5	Mean difference (months): G2: crude: −0.4; adjusted: 0.0 (−3.8, 3.8); G3: crude: 1.1; adjusted: 1.4 (−1.9, 4.9); G4: crude: −0.2; adjusted: 0.5 (−2.5, 3.5); G5: crude: 2.2; adjusted: 2.6 (−2.2, 7.4)
PHV and age at PHV		
(28)	Age at PHV stratified by exposure status and β coefficient for exposure to GDM in utero after adjusting for child’s race/ethnicity	Age at PHV (years) : GDM: 12.68; No GDM: 12.92
		β coefficient and *P*-value not reported (but figure shows overlapping confidence intervals of age at PHV between exposed and the unexposed)
	PHV among exposed and unexposed girls and boys and beta coefficient for exposure to GDM in utero after adjusting for child’s race/ethnicity	PHV (cm/year): GDM: 9.65; No GDM: 9.28; β coefficient : 0.04; *P* < 0.001
Spermarche		
(31)	Mean age at spermarche in sons of mothers without diabetes	Mean age (years): 13.4
	Crude and adjusted (for maternal age at menarche, maternal age at birth, socioeconomic status, cohabitation, parity and maternal BMI) mean monthly difference in age (95% CI) at spermarche	Mean difference (months): crude: 0; adjusted: 0.7 (−2.9, 4.3)
Other pubertal outcomes		
(31)	Mean age at VB, AV, AH, acne in sons of mothers without diabetes	Mean age (years) : VB: 13.0; AV: 15.0; AH: 13.3; Acne: 12.2
	Crude and adjusted (for maternal age at menarche, maternal age at birth, socioeconomic status, cohabitation, parity and maternal BMI) mean monthly difference in age (95% CI) at VB, AV, development of AH and acne	Mean difference (months): VB: crude: −1.8; adjusted: −0.8 (−4.6, 2.8); AV: crude: −3.2; adjusted: −2.5 (−8.4, 3.4); AH: crude: −4.3; adjusted: −2.9 (−7.4, 1.8); Acne: crude: 0.8; adjusted: 1.8 (−2.1, 5.7)

AH, axillary hair; AV, adult voice; HR, hazard ratio; NS, not significant; OR, odds ratio; PHV, peak height velocity; RR, risk ratio; Tanner stage G, Tanner stage genital development; Tanner stage PH, Tanner stage pubic hair; VB, voice break.

#### *Pubic hair development (p*ubarche)

Three studies evaluated maternal GDM and its association with pubarche in their sons (33, 34, 35). Grunnet *et al.* (26) reported 74% increase in the odds of having reached tanner stage ≥PH2 among boys born to mothers with GDM compared to those born to mothers without GDM after adjustment for age (adjusted OR: 1.74 (95% CI: 0.92, 3.28)) (Table 3). Lauridsen *et al.* (30) reported trends to earlier age at public hair stages among boys born to mothers with GDM compared to boys born to mothers without GDM (adjusted mean monthly difference for PH2: −1.4 (95% CI: −5.3, 2.4); PH3: −1.3 (95% CI: −4.6, 1.9); PH4: −0.8 (95% CI: −3.4, 1.6); PH5: −1.7 (95% CI: −4.7, 1.3)) (Table 3). Monteilh *et al.* (31) performed a step-wise inclusion of covariates based on statistical significance to predict age at transition into stages PH2–4. GDM was not included in the analysis for transition into stages PH2 and PH4 due to statistical insignificance at the predictor selection stage of the analysis. In the model predicting transition to stage PH3, GDM was included as a predictor along with either offspring BMI or height and weight anthropometrics measures separately recorded at age 8. In the model with BMI, boys born to GDM exposed mothers showed 2-month advancement in the age at transition to PH3 (Table 3). Median age of transition to PH3 was 12.6 (95% CI: 12.4, 12.7) for boys born to mothers with GDM compared to the entire cohort’s median age of 12.8 (95% CI: 12.7, 12.8). In the model with height and weight anthropometrics instead of BMI, median age of transition to PH3 for boys born to mothers with GDM was 12.8 (95% CI: 12.6, 13.0) compared to the entire cohort’s median age of transition to PH3 13.0 (95% CI: 12.8, 13.1).

#### Genital development and testicular volume

Two studies considered the association between maternal GDM and the age at onset of male genital development (26, 30). Grunnet *et al.* (26) reported genital stage ≥G2 in 63 (32.6%) boys of mothers who had GDM and 66 (37.5%) boys of mothers without GDM; after adjusting for their age, they reported an OR of 1.24 (95% CI: 0.74, 2.14). The same study did not report a similar direction of effect for gonadarche (testicular volume ≥ 4 mL) in boys born to mothers with GDM (adjusted OR: 0.77 (0.42–1.41)) (Table 3). Lauridsen *et al.* (30) did not find any association between maternal GDM and the age at genital stages 2–5 (adjusted mean monthly difference in G2: −0.0 (95% CI: −3.8, 3.8); G3: 1.4 (95% CI: −1.9, 4.9); G4: 0.5 (95% CI: −2.5, 3.5); G5: 2.6 (95% CI: -2.2, 7.4)) (Table 3).

#### Age at peak height velocity

Hockett *et al.* (27) reported age at PHV among boys born to mothers with and without GDM as 12.68 and 12.92 years, respectively, with overlapping confidence intervals. Further, they reported a 4% increased PHV among boys born to mothers with GDM compared to boys born to mothers without GDM after adjusting for race/ethnicity (Table 3).

#### Spermarche

Lauridsen *et al.* (30) studied the association between maternal GDM and age at first ejaculation. The study did not observe any association (adjusted mean monthly difference: 0.7 (−2.9, 4.3)) (Table 3).

## Discussion

To our knowledge, this is the first systematic review that comprehensively explores the relationship between maternal GDM and pubertal timing; also stratified by offspring gender. Although the current evidence is limited, we noted a subtle trend towards earlier pubertal timing in children exposed to maternal hyperglycemia manifested as GDM *in utero*.

We have included studies that report ‘maturational events’ that are considered to define puberty, that is, the development of secondary sexual characteristics such as pubic hair, breast (in girls) and penile growth (in boys), growth parameters (such as PHV and age at PHV) and critical events, such as menarche and spermarche.

The point estimates in all the studies are consistent with an earlier age at onset of pubarche in both boys and girls of mothers with GDM compared to the control population. Notably, there was discrepancy in the offspring sex-specific effect of maternal GDM on pubarche. Specifically, Grunnert *et al.* (26) suggest more pronounced GDM-related odds of pubarche in boys compared to girls while Lauridsen *et al.* (30) report a more pronounced GDM-related precocity of all pubic hair stages in girls compared to boys.

Four studies that examined the onset of breast development (26, 28, 29, 30) and two studies that examined menarche (25, 30) showed variations in the direction, strength and significance of association with maternal GDM. The timing of genital growth and spermarche did not appear to be affected in boys born to mothers with GDM (26, 30). One study did collect information on genital development but due to invalidation of longitudinal recording indicated by a significant proportion of boys proposing Tanner stage regression, this outcome was not analysed (31). Growth parameters such as PHV and age at PHV in boys and girls were associated with maternal GDM (27).

Although the present evidence suggests that maternal GDM might be related to early pubertal timing in their offspring, this effect is rather modest or not evident in the full range of pubertal ‘maturational events’, suggesting a complex interplay between GDM and puberty.

Previous studies have suggested a relationship between maternal GDM and offspring adiposity (32). Adiposity and ‘over-nutrition’ can be considered predictors of pubertal timing and principal determinants for the initiation and maintenance of pubertal maturational events (33), hence, the association between maternal GDM and offspring pubertal timing could be mediated by offspring adiposity and pre-adolescence BMI. This is supported by the analysis by Hockett *et al.* (27), in which the association between maternal GDM and age at PHV is attenuated by adjustment for offspring BMI z-score.

Several studies have suggested a negative association between pre-pregnancy BMI and timing of puberty (34, 35). High pre-pregnancy BMI is an established risk factor of GDM (36); however, considering the available studies it is difficult to dissect the effects of GDM and pre-pregnancy BMI on offspring pubertal timing. Furthermore, an U-shaped association between age at menarche and future risk of GDM has been established (37). Therefore, it is plausible that a synergistic effect exists between the intrauterine effect of hyperglycemia on pubertal timing in the offspring and the genetic influence of earlier maternal age at menarche. In addition to the already explored factors adjusted for in various studies, several other factors such as birthweight (both higher and lower) (38, 39), exogenous exposure to endocrine-disrupting chemicals such as phthalates, pesticides and bisphenol A in the mother-offspring home environment (40, 41) could have confounded this association. The same applies to leptin, which largely correlates with body fat content. Higher plasma leptin levels have been documented in GDM (42) and may contribute to gestational programming of offspring obesity as leptin is regarded as a permissive signal for puberty initiation (43).

Trends towards earlier pubarche is probably one of the most consistent precocities of all puberty parameters assessed by the studies analysed in this review. It is important to note that the rise of adrenal androgen production in late childhood contributes to the development of pubic (and axillary) hair, an event known as adrenarche (44, 45). Adrenarche is a phenomenon currently not well understood, but not related and in fact strictly independent of gonadarche. As adrenarche and gonadarche frequently overlap, it is clinically not possible to distinguish if pubarche is caused by adrenal or testicular androgens in boys, however, it is likely that pubic hair develops as a consequence of adrenal androgen action in girls. Premature adrenarche has been traditionally regarded as benign variant of normal ‘puberty’, however, there is some evidence suggesting that children with premature adrenarche have metabolic dysfunction, in particular abnormal glucose metabolism (46).

To assess the dynamics of pubertal development accurately is difficult, both in the individual clinical setting but even more so based on observational studies. Tanner staging is an unequivocally accepted clinical tool to assess pubertal milestones (47), but prone to inter-observer differences (48, 49) and over/underestimation of Tanner staging frequently occurs when being self-reported (50). Assessment of the activation of the hypothalamic–pituitary–gonadal axis via LHRH stimulation testing or overnight LH sampling as an outcome measure would aid in objectification as well as differentiation of central and peripheral causes for advancement in pubertal timing (51, 52), albeit difficult to perform in larger study populations due to invasiveness, logistics and cost implications. Rare underlying sinister pathologies, such as sex steroid-producing tumours or hypothalamic abnormalities, can affect pubertal timing, however, were only systematically excluded in one of the studies (29).

The findings of the present review should be interpreted in the context of its limitations. One of them was the wide variation in the sample sizes of the included studies. However, it should be noted that no correlation was observed between the sample size and the magnitude or significance of effect estimates. Two pairs of derived their cohorts from the same databases (26, 28, 29, 30), suggesting a possible overlap of the participants between these pairs of studies.

The summary measures were widely heterogeneous across all of the studies, preventing any meaningful attempt to statistically pool the results. The interval spanned between subsequent observations of Tanner stages or anthropometrics varied across the included longitudinal studies, ranging between 6 months and 1.5 years. Also, there was a high percentage of children who did not agree to report their Tanner stage, which could bias the effect estimates as previous studies report an association between Tanner stage of children and their agreement to have it recorded (53). Therefore, interval and informative censoring embedded in the observational nature of the included studies were potential limitations in accurately discerning the association between maternal GDM and pubertal timing of children. Lastly, both the diagnostic criteria and the approach to testing for GDM differ widely by country, from no routine to universal screening (54). Routine screening has been recommended by the Diabetes in Pregnancy Study Groups (IADPSG) after results from the hyperglycaemia and adverse pregnancy outcomes (HAPO) study were published in 2008 (55). Since screening practices have changed over time with a trend to test and diagnose more comprehensively in recent years, a shift towards milder GDM phenotypes has been observed (56). In the studies included in this review, GDM was diagnosed between 1991 and 2006 based on different diagnostic criteria (Table 1), and it is possible that those differences together with a change of screening practices over time contribute to a larger heterogeneity in the reported associations with offspring’s pubertal outcome measures.

In order to strengthen the evidence base for the association between maternal GDM and pubertal timing, large-scale prospective cohort studies should be conducted, ideally with standardized approaches in diagnosing GDM and recording of wide range of confounders at baseline. Future research is needed to understand the biological link between the maternal–fetal endocrine system. This can help in the identification of potential interventions to limit the progression of a potential transgenerational continuum of endocrine disturbance and adverse effects on metabolic health.

## Supplementary Material

Supplementary Table 1: Search Strategy

Supplementary Table 2: Template data extraction form

Supplementary Table 3: Template risk of bias assessment form

Supplementary Table 4: PRISMA Checklist

## Declaration of interest

Wiebke Arlt is the Editor-in-Chief of EJE. Wiebke Arlt was not involved in the review or editorial process for this paper, on which she is listed as an author.

## Funding

This work was supported by the Academy of Medical Scienceshttp://dx.doi.org/10.13039/501100000691 (Starter Grant for Clinical Lecturers SGL020/1013, to J I) and the Wellcome Trusthttp://dx.doi.org/10.13039/501100009053 (Investigator Grant WT209492/Z/17/Z, to W A). K N is a UK Research and Innovation (UKRI)/Health Data Research (HDR) UK Innovation Clinical Fellow. W A receives support from the National Institute for Health Researchhttp://dx.doi.org/10.13039/501100000272 (NIHR) Birmingham Biomedical Research Centre at the University Hospitals Birmingham NHS Foundation Trusthttp://dx.doi.org/10.13039/100013963 and the University of Birminghamhttp://dx.doi.org/10.13039/501100000855 (Grant BRC-1215-20009). The views expressed are those of the authors and not necessarily those of the NIHR or the Department of Health and Social Care UK. The funders of the study had no role in the: design and conduct of the study; collection, management, analysis, and interpretation of the data; preparation, review, or approval of the manuscript; decision to submit the manuscript for publication.
