# Supplementary material for: Pubertal timing in boys and girls born to mothers with gestational diabetes mellitus: a systematic review
Source: Eur J Endocrinol. 2020 Oct 8;184(1):51–64. doi: 10.1530/EJE-20-0296 (PMC7707806; doi:10.1530/EJE-20-0296)
Supplement: Supplementary Table 1: Search Strategy [file supplementary_table_1.pdf]

Supplementary Table 1: Search Strategy

Search strategy involved combining the search terms of gestational diabetes mellitus and precocious puberty with the 'AND' Boolean operator.

**Search terms for Gestational Diabetes Mellitus**	
1.	gestational diabetes.ti,ab.
2.	exp Diabetes, Gestational/
3.	GDM.ti,ab.
4.	((gestation$ or pregnan$) adj3 (diabetes or diabetic or glucose intolerance or impaired glucose tolerance or hyperglycaemia or glycaemic index or hyperglycemia or glycemic index)).ti,ab.
5.	1 or 2 or 3 or 4
**Search terms to select precocious puberty**	
1.	Puberty/
2.	((timing or time) adj5 pubert$).ti,ab.
3.	sexual maturation.ti,ab. or Sexual Maturation/
4.	((premature or precocious or early) adj5 pubert$).ti,ab.
5.	Puberty, Precocious/ or sexual precocity.ti,ab.
6.	sexual prematurity.ti,ab.
7.	pubarche.ti,ab.
8.	axillarche.ti,ab.
9.	((axillary or pubic) adj2 hair).ti,ab.
10.	thelarche.ti,ab.
11.	(breast adj3 develop$).ti,ab.
12.	menarche.ti,ab. or exp Menarche/
13.	testicular volume.ti,ab.
14.	first spermatorrhea.ti,ab.
15.	spermarche.ti,ab.
16.	(tanner and (scale or score)).ti,ab.
17.	(age$ adj4 takeoff).ti,ab.
18.	Adolescent Development/
19.	(Growth/ or growth.ti,ab.) and spurt.ti,ab
20.	(peak height velocity or PHV).ti,ab.
21.	1 or 2 or 3 or 4 or 5 or 6 or 7 or 8 or 9 or 10 or 11 or 12 or 13 or 14 or 15 or 16 or 17 or 18 or 19 or 20
