# Supplementary material for: Pubertal timing in boys and girls born to mothers with gestational diabetes mellitus: a systematic review
Source: Eur J Endocrinol. 2020 Oct 8;184(1):51–64. doi: 10.1530/EJE-20-0296 (PMC7707806; doi:10.1530/EJE-20-0296)
Supplement: Supplementary Table 2: Template data extraction form [file supplementary_table_2.pdf]

Supplementary Table 2: Template data extraction form

Study ID	Basic information	Participants	Maternal and offspring characteristics	Follow-up	Outcomes
Maternal diabetes mellitus and timing of pubertal development in daughters and sons: a nationwide cohort study. Lea L.B.Lauridsen et al., July 2018	*Study design: Retrospective Cohort Study*<br><br>*Language: English*<br><br>*Location: Denmark*<br><br>*Study period: May 2012 - .*<br><br>*Funding and conflict of interest: None declared*	*Setting:*<br>1) *Mother Cohort: The Danish National Birth Cohort (DNBC)*<br>2) *Offspring cohort: Puberty Cohort*<br><br>*Method of participant recruitment: Recruited at the first antenatal visit at their GP between week 6 and 12 of gestation*<br><br>*Eligibility criteria:*<br>1) *Mothers: mothers had to have answered the first interview during*	*Maternal Characteristics:*<br>1) *Age at delivery: in years; mean(95% CI)*<br>*GDM – 31.9 (31.4-32.5)*<br>*Without diabetes – 30.6 (30.5-30.7)*<br><br>2) *Parity:*<br>*GDM –*<br>*First child – 123 (48.6)*<br>*Second or more child – 130 (51.4)*<br>*Without diabetes –*<br>*First child – 7731 (50.5)*<br>*Second or more child – 7589 (49.5)*<br><br>3) *Pre-pregnancy BMI (kg/m^2) –*<br>*GDM –*<br>*<18.5 kg/m^2 – 3 (1.2)*<br>*18.5 - <25 kg/m^2 – 93 (37.4)*<br>*25 - <30 kg/m^2 – 64 (25.7)*<br>*<30 kg/m^2 – 89 (35.7)*<br>*Without diabetes -*	*Exposure ascertainment method and criteria: Combination of register-based and self-reported questionnaire*<br>➔ *Based on ICD codes from Danish National Patient Registry (DNPR)*<br>➔ *two telephone interviews during pregnancy and the one telephone interview 6 months postpartum, the mothers also provided self-reported information on diabetes.*<br><br>*In case of discrepancy between diabetes type in the DNPR and the DNB, DNPR was used*	*Outcome ascertainment methods:*<br><br>*Self-reported outcome measures fromtelephone interviews*<br><br>*Outcome metrics: adjusted mean monthly difference in age of daughters born to women with GDM and women without diabetes*<br>***In daughters:***<br>*Tanner Stage*<br>*Pubic hair*<br>*Stage 2: -4.8 (-7.7, -2.0)*<br>*Stage 3: -2.2 (-4.4, 0)*<br>*Stage 4: -1.6 (-4.8, 1.6)*<br>*Stage 5:-6.0 (-10.8, -1.2)*<br><br>*Breast development:*<br>*Stage 2: -4.6 (-10.1, 1.0)*<br>*Stage 3: -1.9 (-5.0, 1.2)*<br>*Stage 4:-0.5 (-3.2, 2.4)*<br>*Stage 5:-1.8 (-7.9, 4.3)*

Study ID	Basic information	Participants	Maternal and offspring characteristics	Follow-up	Outcomes
		pregnancy and not withdrawn their consent of participation before initiation of the Puberty Cohort in May2012<br>2) Offsprings: live-born singleton children; Answered at least one questionnaire in the puberty cohort or the 11-year follow-up questionnaire for the DNBC cohort<br>Exclude criteria:<br>1) Mother:<br>2) Offsprings: no information on pubertal	<18.5 kg/m^2 – 1050 (7.0)<br>18.5 - <25 kg/m^2 – 9435 (62.4)<br>25 - <30 kg/m^2 – 3176 (21.0)<br><30 kg/m^2 – 1452 (9.6)<br><br>4) Pregnancy weight-gain – NM<br>5) BMI during pregnancy: n(%) – NM<br>6) Fasting blood glucose - NM<br>7) Insulin use during pregnancy – In Denmark, all pregnant women with severe T2DM or GDM will be treated with insulin<br>8) Maternal age at menarche –<br>GDM –<br>Earlier than peers – 93 (36.8)<br>Same as peers – 122 (48.2)<br>Later than peers – 38 (15.0)<br>Without diabetes –	Gestational age at study entry for mothers:<br>6 and 12 of gestation<br><br>Length of follow-up:<br>until the offspring reached full sexual maturation (pubertal Tanner stage 5 in both pubic hair [sons and daughters] and breast development [daughters]or genital [sons])(22, 23)or turned 18 years,<br><br>Loss to follow-up:<br>Only 70.51% percent of the children had information on pubertal development. The rest were excluded from the study<br><br>Data collection:<br>Electronic Health Records<br>Telephone interviews<br><br>Statistical method:<br>Data on age at attaining the pubertal milestones were interval, left, or right	Menarche: -2.5 (-4.9, 0.0)<br>Axillary Hair: -3.6 (-7.3, 0.1)<br>Acne: -2.6 (-6.8, 1.6)<br><br>**In sons:**<br>Tanner Stage<br>Pubic hair<br>Stage 2: -1.4 (-5.3, 2.4)<br>Stage 3: -1.3 (-4.6, 1.9)<br>Stage 4: -0.8 (-3.4, 1.6)<br>Stage 5:-1.7 (-4.7, 1.3)<br><br>Genitals development:<br>Stage 2: 0.0 (-3.8, 3.8)<br>Stage 3: 1.4 (-1.9, 4.9)<br>Stage 4:0.5 (-2.5, 3.5)<br>Stage 5:2.6 (-2.2, 7.4)<br><br>Ejaculation: -0.7 (-2.9, 4.3)<br>Voice break: -0.8 (-4.6, 2.8)<br>Adult voice: -2.5 (-8.4, 3.4)<br>Axillary Hair: -2.9 (-7.4, 1.8)<br>Acne: 1.8 (-2.1, 5.7)<br><br>The effect of having BMI >=25 kg/m2and GDM on the pubertal development

Study ID	Basic information	Participants	Maternal and offspring characteristics	Follow-up	Outcomes
		development<br><br>*Number of mother-offspring pairs: n= 15,573*<br>*GDM mother-offspring pairs: n= 253*<br>*[Girls: 122; Boys: 130]*	*Earlier than peers – 3849 (25.3)*<br>*Same as peers – 8732 (57.5)*<br>*Later than peers – 2617 (17.2)*<br><br>*9) Ethnicity - NM*<br><br>*10) Socio-economic status: GDM –*<br>*High grade professional – 48 (19.0)*<br>*Low grade professional – 76 (30.0)*<br>*Skilled worker – 82 (32.4)*<br>*Unskilled worker – 38 (15.0)*<br>*Student – 6 (2.4)*<br>*Economically inactive -3 (1.2)*<br>*Without diabetes –*<br>*High grade professional – 3590 (23.5)*<br>*Low grade professional – 5041 (33.0)*<br>*Skilled worker – 4195 (27.4)*<br>*Unskilled worker – 2079 (13.6)*<br>*Student – 299 (2.0)*	*censored. Data were analysed by a multivariable interval censored regression model for normally distributed time-to-event data using the Stata 15.0 software (Stata Corporation) intreg package. We estimated the crude and adjusted mean monthly difference (with 95% confidence interval [CI]) in age at attaining the pubertal milestones between children of mothers with GDM, and children of mothers without diabetes.*<br>*To take the sampling into account, we applied sampling weights defined as the inverse probability of being sampled. The probability of being sampled was calculated based on the sampling fractions for the 27 sampling groups as well as the reference sample. In*	*was comparable with the sum of the effect of having BMI >25 kg/m2 and not having GDM and the effect of having BMI<25 kg/m2and GDM—indicating an additive effect of overweight on the effect of GDM*<br><br>*List of confounding factors considered: Adjusted for maternal age at menarche (earlier than peers, same time as peers [reference]or later than peers), maternal age at delivery in years (cubic splines with four knots), parity (first child [reference], second child or more), cohabitation of parents (live together [reference], do not live together),highest social class of parents(high grade professional [reference], low grade professional ,skilled worker, unskilled worker, student, economically*

Study ID	Basic information	Participants	Maternal and offspring characteristics	Follow-up	Outcomes
			Economically inactive – 86 (0.6)<br><br>*11) drug/tobacco/alcohol/caffeine use during or prior to the pregnancy : NM*<br>*12) Presence of co-existing comorbid conditions - NM*<br><br>*Offspring characteristics:*<br>*1) Birthweight: in grams; mean(SD)*<br>*GDM – 3648.8 (3569.5 - 3728.2)*<br>*Without diabetes – 3521.9 (3512.4 - 3531.5)*<br>*2) Gestational age at birth – Birth before gestation week 37*<br>*GDM – 25 (9.9)*<br>*Without diabetes – 1054 (6.9)*<br>*3) Anthropometrics of the offspring at the time of outcome measurement - NM*	*addition, we used inverse probability weights to account for nonparticipation in the Puberty Cohort(26). Based on an a priori-specified directed acyclic graph of selection mechanisms in the Puberty Cohort, we estimated the probability of participation for each participant using multivariable logistic regression. The inverse of the predicted probability was used as selection weights. We applied robust s standard errors to account for the weighting and clustering of siblings.*<br><br>*We investigated the role of maternal BMI on the possible association between GDM and pubertal development in daughters and sons, separately, using a joint inter-action analysis.*	*inactive and maternal pre-pregnancy body mass index (BMI) (<18.5,18.5–<25 [reference], 25–<30,R30 kg/m2*
