# Supplementary material for: Pubertal timing in boys and girls born to mothers with gestational diabetes mellitus: a systematic review
Source: Eur J Endocrinol. 2020 Oct 8;184(1):51–64. doi: 10.1530/EJE-20-0296 (PMC7707806; doi:10.1530/EJE-20-0296)
Supplement: Supplementary Table 3: Template risk of bias assessment form [file supplementary_table_3.pdf]

## Supplementary Table 3: Template risk of bias assessment form

**Study ID: Lauridsen et al., 2018**

| Item | Considerations | Authors' judgement | Support for judgement |
|---|---|---|---|
| Representativeness of the GDM cohort | • the subjects covered in this study could be sufficiently different from your population to cause concern<br>• your local setting is likely to differ much from that of the study | Low risk | DNBC cohort |
| Unexposed cohort drawn from the same population | • were all the subjects drawn from the same base cohort? | Low risk | same cohort for control selection |
| Objective ascertainment of exposure | • did they use subjective or objective measurements<br>• were all the subjects classified into exposure groups using the same procedure | Low risk | register based + self-reported information |
| No contamination of exposure status with pre-existing diabetes | • do the measurements truly reflect what you want them to (have they been validated)<br>• does the exposed and the unexposed cohort exclude patients with pre-existing diabetes | Low risk | clear stratification of T1DM, T2DM and GDM |
| Adjustment for covariates in the analysis | • do the authors identify and adjust for confounders in their analysis | Low risk | adjusted for maternal age at menarche, maternal age at birth,socioeconomic staus, cohabitation, parity and maternal pre-pregnancy BMI |
| Blind outcome measurement | • were the subjects and/or the outcome assessor blinded to exposure (does this matter) | High risk | no report of blinding of the researchers |
| Objective ascertainment of outcomes | • were the measurement methods similar in the different groups | High risk | questionnaire and self-reports |

| | | | |
|---|---|---|---|
| | • did they use subjective or objective measurements<br>• do the outcomes truly reflect what you want them to (have they been validated) | | |
| Imprecision of outcome timing accounted for in the analysis | • were the age at maturation events known exactly<br>• If not, did the authors use appropriate statistical approach such as interval censored regression analysis for time-to-event data / Did the authors consider multiple longitudinal outcome measurements | Low risk | interval censoring |
| Other causes of precocious puberty investigated | • did the authors record and report non-idiopathic causes of sex steroid producing tumours or hypothalamic abnormalities. | High risk | no report |
| Sufficient follow-up time | • was the follow-up of the offspring long enough for the range of maturational events to be recorded?<br>• did the authors report median follow-up period? | Low risk | from the age of 11.5, children were followed up until they all they reached full sexual maturation or turned 18 |
| Loss to follow-up minimal or nil | • proportion willing to report outcomes<br>• proportion dropped out of the cohort | High risk | only 71% of the children provided information on pubertal development |
